# Supplementary material for: The Effect of Exercise and Nutritional Interventions on Body Composition in Patients with Advanced or Metastatic Cancer: A Systematic Review
Source: Nutrients. 2022 May 18;14(10):2110. doi: 10.3390/nu14102110 (PMC9145470; doi:10.3390/nu14102110)
Supplement: Supplementary file 1 [file nutrients-14-02110-s001.zip › nutrients-1720608-supplementary.pdf]

Supplementary Material

Supplementary Table S1. Full search strategy

[1] PubMed

Search	Query	Results
#1	"metastatic"[tiab] OR "metastasis" OR "Metastases" [tiab] OR "advanced" [tiab] OR "advanced-stage" [tiab] OR "palliative" [tiab] OR "palliative care" [tiab] OR "incurable" [tiab] OR "End of life" [tiab] OR "stage III" [tiab] OR "stage IV"[tiab] OR "inoperable"[tiab] OR "neoplasm metastasis"[mh] OR "palliative care"[mh]	1175275
#2	"Neoplasms"[tiab] OR "neoplasm"[tiab] OR "cancer"[tiab] OR "carcinoma"[tiab] OR "tumor"[tiab] OR "tumour"[tiab] OR "malignancy"[tiab] OR "malignant"[tiab] OR "solid tumor"[tiab] OR "lymphoma"[tiab] OR "leukaemia"[tiab] OR "leukemia"[tiab] OR "myeloma"[tiab] OR "haematological malignancy"[tiab] OR "hematological malignancy"[tiab] OR "neoplasms"[mh]	4695854
#3	"Exercise"[tiab] OR "exercise therapy"[tiab] OR "exercises"[tiab] OR "physical activity"[tiab] OR "physical activities"[tiab] OR "Endurance Training"[tiab] OR "aerobic training"[tiab] OR "Resistance Training"[tiab] OR "resistance training"[tiab] OR "strength training"[tiab] OR "Weight Lifting"[tiab] OR "weightlifting"[tiab] OR "Physical Conditioning, Human"[tiab] OR "physical conditioning"[tiab] OR "Muscle Contraction"[tiab] OR "Circuit-Based Exercise"[tiab] OR "circuit training"[tiab] OR "High-Intensity Interval Training"[tiab] OR "interval training"[tiab] OR "Running"[tiab] OR "Jogging"[tiab] OR "Bicycling"[tiab] OR "Swimming"[tiab] OR "Walking"[tiab] OR "Sports"[tiab] OR "sport"[tiab] OR "Yoga"[tiab] OR "Tai Ji"[tiab] OR "tai ji"[tiab] OR "tai chi"[tiab] OR "exercise"[mh] OR "physical exertion"[mh] OR "physical fitness"[mh]	719171
#4	"body composition" or "muscle" or "fat" or "weight" or "BMI" or "sarcopenia" or "mass" or "lean" or "bone" or "dexa" or "DXA" or "Whole-body dual-energy x-ray absorptiometry" or "BIA" or "bioelectrical impedance assessment" or "cachexia" or "nutritional status" or "fat percentage" or "nutrition" or "Kg" or ("physical" and "outcome")	4617766
#5	#1 AND #2 AND #3 AND #4	1520
#6	Animals[mh] NOT humans [mh]	4960809
#7	#5 NOT #6	1464

[2] Excerpta Medica Database [EMBASE]

Search	Query	Results
1	(metastatic or Metastases or advanced or advanced-stage or palliative or incurable or stage III or stage IV or inoperable).mp. [mp=title, abstract, heading word, drug trade name, original title, device manufacturer, drug manufacturer, device trade name, keyword heading word, floating subheading word, candidate term word]	1440499
2	metastasis/ or *"cancer of unknown primary site"/ or *central nervous system metastasis/ or *digestive system metastasis/ or *distant metastasis/ or *endocrine system metastasis/ or *experimental metastasis/ or *"head and neck metastasis"/ or *hematopoietic system metastasis/ or *in-transit metastasis/ or	335324

	*local metastasis/ or *locomotor system metastasis/ or *lymphatic system metastasis/ or *metastatic breast cancer/ or *metastatic colon cancer/ or *metastatic melanoma/ or *micrometastasis/ or *pancreas metastasis/ or *peritoneum metastasis/ or *port site metastasis/ or *regional metastasis/ or *satellite metastasis/ or *sister mary joseph nodule/ or *skin metastasis/ or *soft tissue metastasis/ or *thoracic cavity metastasis/ or *urogenital tract metastasis/ or *visceral metastasis/	
3	advanced cancer/	135570
4	(Neoplasms or neoplasm or cancer or carcinoma or tumor or tumour or malignancy or malignant or solid tumor or lymphoma or leukaemia or leukemia or myeloma or haematological malignancy or hematological malignancy).mp. [mp=title, abstract, heading word, drug trade name, original title, device manufacturer, drug manufacturer, device trade name, keyword heading word, floating subheading word, candidate term word]	6112558
5	neoplasm/ or malignant neoplasm/ or metastasis/ or mixed tumor/ or "neoplasms of uncertain behavior"/ or neoplasms subdivided by anatomical site/ or radiation induced neoplasm/	773175
6	(Exercise or exercise therapy or exercises or physical activity or physical activities or Endurance Training or aerobic training or Resistance Training or resistance training or strength training or Weight Lifting or weightlifting or Physical Conditioning, Human or physical conditioning or Muscle Contraction or Circuit-Based Exercise or circuit training or High-Intensity Interval Training or interval training or Running or Jogging or Bicycling or Swimming or Walking or Sports or sport or Yoga or Tai Ji or tai ji or tai chi).mp. [mp=title, abstract, heading word, drug trade name, original title, device manufacturer, drug manufacturer, device trade name, keyword heading word, floating subheading word, candidate term word]	1131836
7	exp exercise/	382755
8	(body composition or muscle or fat or weight or BMI or sarcopenia or mass or lean or bone or dexa or DXA or Whole-body dual-energy x-ray absorptiometry or BIA or bioelectrical impedance assessment or cachexia or nutritional status or fat percentage or nutrition or Kg).mp. [mp=title, abstract, heading word, drug trade name, original title, device manufacturer, drug manufacturer, device trade name, keyword heading word, floating subheading word, candidate term word]	6949962
9	body composition/ or body adiposity index/ or body distribution/ or body fat/ or body fat distribution/ or body fat percentage/ or body water/ or total body fat/	111353
10	muscle mass/	31937
11	fat mass/	19372
12	1 or 2 or 3	1565315
13	4 or 5	6119564
14	6 or 7	1134595

15	8 or 9 or 10 or 11	6953636
16	12 and 13 and 14	8401
17	limit 16 to (abstracts and english language)	7440
18	17 and 15	3774
19	limit 18 to english language	3774
20	("case report" or "review" or "meta analysis" or "observational").m_titl. [mp=title, abstract, heading word, drug trade name, original title, device manufacturer, drug manufacturer, device trade name, keyword heading word, floating subheading word, candidate term word]	1079219
21	19 not 20	3468

[3] Cumulative Index to Nursing and Allied Health Literature [CINAHL]

Query	Terms	Results
#1	TI (“metastatic” OR “metastasis” OR “Metastases” OR “advanced” OR “advanced-stage” OR “palliative” OR “palliative care” OR “incurable” OR “End of life” OR “stage III” OR “stage IV” OR “inoperable” OR (MH“neoplasm metastasis+”)) OR AB (“metastatic” OR “metastasis” OR “Metastases” OR “advanced” OR “advanced-stage” OR “palliative” OR “palliative care” OR “incurable” OR “End of life” OR “stage III” OR “stage IV” OR “inoperable” OR (MH“neoplasm metastasis+”)) OR MW (“metastatic” OR “metastasis” OR “Metastases” OR “advanced” OR “advanced-stage” OR “palliative” OR “palliative care” OR “incurable” OR “End of life” OR “stage III” OR “stage IV” OR “inoperable” OR (MH“neoplasm metastasis+”))	283,043
#2	TI (“Neoplasms" OR “neoplasm” OR “cancer” OR “carcinoma” OR “tumor” OR “tumour” OR “malignancy” OR “malignant” OR “solid tumor” OR “lymphoma” OR “leukaemia” OR “leukemia” OR “myeloma” OR “haematological malignancy” OR “hematological malignancy” OR (MH “neoplasms”) OR (MH “carcinoma+”) OR (MH “lymphoma+”) OR (MH “leukemia+”) OR (MH “Neoplasms by histologic type+”) OR “Neoplasms by site+”)) OR AB (“Neoplasms" OR “neoplasm” OR “cancer” OR “carcinoma” OR “tumor” OR “tumour” OR “malignancy” OR “malignant” OR “solid tumor” OR “lymphoma” OR “leukaemia” OR “leukemia” OR “myeloma” OR “haematological malignancy” OR “hematological malignancy” OR (MH “neoplasms”) OR (MH “carcinoma+”) OR (MH “lymphoma+”) OR (MH “leukemia+”) OR (MH “Neoplasms by histologic type+”) OR “Neoplasms by site+”)) OR MW (“Neoplasms" OR “neoplasm” OR “cancer” OR “carcinoma” OR “tumor” OR “tumour” OR “malignancy” OR “malignant” OR “solid tumor” OR “lymphoma” OR “leukaemia” OR “leukemia” OR “myeloma” OR “haematological malignancy” OR “hematological malignancy” OR (MH “neoplasms”) OR (MH “carcinoma+”) OR (MH “lymphoma+”) OR (MH “leukemia+”) OR (MH “Neoplasms by histologic type+”) OR “Neoplasms by site+”))	818,698
#3	TI (“Exercise” OR “exercise therapy ” OR “exercises” OR “physical activity” OR “physical activities” OR “Endurance Training” OR “aerobic training” OR “Resistance Training” OR “resistance training” OR “strength training” OR “Weight Lifting” OR “weightlifting” OR “Physical Conditioning, Human” OR “physical conditioning” OR “Muscle Contraction” OR OR “Circuit-Based Exercise” OR “circuit training” OR “High-Intensity Interval Training” OR	381,139

	"interval training" OR "Running" OR "Jogging" OR "Bicycling" OR "Swimming" OR OR "Walking" OR "Sports" OR "sport" OR "Yoga" OR "Tai Ji" OR "tai ji" OR "tai chi" OR (MH "exercise+") OR (MH "therapeutic exercise+") OR (MH "functional training+") OR (MH "athletic training") OR (MH "sports+")) OR AB ("Exercise" OR "exercise therapy " OR "exercises" OR "physical activity" OR "physical activities" OR "Endurance Training" OR "aerobic training" OR "Resistance Training" OR "resistance training" OR "strength training" OR "Weight Lifting" OR "weightlifting" OR "Physical Conditioning, Human" OR "physical conditioning" OR "Muscle Contraction" OR OR "Circuit-Based Exercise" OR "circuit training" OR "High-Intensity Interval Training" OR "interval training" OR "Running" OR "Jogging" OR "Bicycling" OR "Swimming" OR OR "Walking" OR "Sports" OR "sport" OR "Yoga" OR "Tai Ji" OR "tai ji" OR "tai chi" OR (MH "exercise+") OR (MH "therapeutic exercise+") OR (MH "functional training+") OR (MH "athletic training") OR (MH "sports+")) OR MW ("Exercise" OR "exercise therapy " OR "exercises" OR "physical activity" OR "physical activities" OR "Endurance Training" OR "aerobic training" OR "Resistance Training" OR "resistance training" OR "strength training" OR "Weight Lifting" OR "weightlifting" OR "Physical Conditioning, Human" OR "physical conditioning" OR "Muscle Contraction" OR OR "Circuit-Based Exercise" OR "circuit training" OR "High-Intensity Interval Training" OR "interval training" OR "Running" OR "Jogging" OR "Bicycling" OR "Swimming" OR OR "Walking" OR "Sports" OR "sport" OR "Yoga" OR "Tai Ji" OR "tai ji" OR "tai chi" OR (MH "exercise+") OR (MH "therapeutic exercise+") OR (MH "functional training+") OR (MH "athletic training") OR (MH "sports+"))	
#4	TI ("body composition" or "muscle" or "fat" or "weight" or "BMI" or "sarcopenia" or "mass" or "lean" or "bone" or "dexa" or "DXA" or "Whole-body dual-energy x-ray absorptiometry" or "BIA" or "bioelectrical impedance assessment" or "cachexia" or "nutritional status" or "fat percentage" or "nutrition" or "Kg" or (MH "body composition+")) OR AB ("body composition" or "muscle" or "fat" or "weight" or "BMI" or "sarcopenia" or "mass" or "lean" or "bone" or "dexa" or "DXA" or "Whole-body dual-energy x-ray absorptiometry" or "BIA" or "bioelectrical impedance assessment" or "cachexia" or "nutritional status" or "fat percentage" or "nutrition" or "Kg" or (MH "body composition+")) OR MW ("body composition" or "muscle" or "fat" or "weight" or "BMI" or "sarcopenia" or "mass" or "lean" or "bone" or "dexa" or "DXA" or "Whole-body dual-energy x-ray absorptiometry" or "BIA" or "bioelectrical impedance assessment" or "cachexia" or "nutritional status" or "fat percentage" or "nutrition" or "Kg" or (MH "body composition+"))	811,358
#5	MJ (MH "Animals+") OR (MH "birds+") OR (MH "fish+") (MH "rodents+") NOT "human"	103,017
#6	(S1 and S2 and S3 and S4) NOT S5	423

[4] Cochrane Central Register of Controlled Trials [CENTRAL]

Query	Terms	Results
#1	("metastatic"):ti,ab OR ("metastasis"):ti,ab OR ("Metastases"):ti,ab OR ("advanced"):ti,ab OR ("advanced-stage"):ti,ab OR ("palliative"):ti,ab OR	99130

	(“palliativecare”):ti,ab OR (“incurable”):ti,ab OR (“Endoflife”):ti,ab OR (“stageIII”):ti,ab OR (“stageIV”):ti,ab OR (“inoperable”):ti,ab	
#2	MeSH descriptor: [Neoplasm Metastasis] explode all trees	5413
#3	MeSH descriptor: [Palliative Care] explode all trees	1742
#4	#1 or #2 or #3	101056
#5	("neoplasms"):ti,ab OR (“neoplasm”):ti,ab OR (“cancer”):ti,ab OR (“carcinoma”):ti,ab OR (“tumor”):ti,ab OR (“tumour”):ti,ab OR (“malignancy”):ti,ab OR (“malignant”):ti,ab OR (“solidtumor”):ti,ab OR (“lymphoma”):ti,ab OR (“leukaemia”):ti,ab OR (“leukemia”):ti,ab OR (“myeloma”):ti,ab OR (“haematologicalmalignancy”):ti,ab OR (“hematologicalmalignancy”):ti,ab	221184
#6	MeSH descriptor: [Neoplasms] this term only	6710
#7	MeSH descriptor: [Neoplasms by Histologic Type] explode all trees	31930
#8	MeSH descriptor: [Neoplasms by site] explode all trees	64311
#9	#5 or #6 or #7 or #8	233957
#10	(“Exercise”):ti,ab OR (“exercisetherapy”):ti,ab OR (“exercises”):ti,ab OR (“physicalactivity”):ti,ab OR (“physicalactivities”):ti,ab OR (“EnduranceTraining”):ti,ab OR (“aerobictraining”):ti,ab OR (“ResistanceTraining”):ti,ab OR (“resistancetraining”):ti,ab OR (“strengthtraining”):ti,ab OR (“WeightLifting”):ti,ab OR (“weightlifting”):ti,ab OR (“PhysicalConditioning,Human”):ti,ab OR (“physicalconditioning”):ti,ab OR (“MuscleContraction”):ti,ab OR (“Circuit-BasedExercise”):ti,ab OR (“circuittraining”):ti,ab OR (“High-IntensityIntervalTraining”):ti,ab OR (“intervaltraining”):ti,ab OR (“Running”):ti,ab OR (“Jogging”):ti,ab OR (“Bicycling”):ti,ab OR (“Swimming”):ti,ab OR (“Walking”):ti,ab OR (“Sports”):ti,ab OR (“Sport”):ti,ab OR (“Yoga”):ti,ab OR (“Tai ji”):ti,ab OR (“tai ji”):ti,ab OR (“tai chi”):ti,ab	144114
#11	MeSH descriptor: [Exercise] explode all trees	27529
#12	MeSH descriptor: [Physical Exertion] this term only	3949
#13	MeSH descriptor: [Sports] this term only	16826
#14	#10 or #11 or #12 or #13	148924
#15	“bodycomposition” OR “muscle” OR “fat” OR “weight” OR “BMI” OR “sarcopenia” OR “mass” OR “lean” OR “bone” OR “dexa” OR “DXA” OR	426970

	"Whole-body dual-energy x-ray absorptiometry" OR "BIA" OR "bioelectrical impedance assessment" OR "cachexia" OR "nutritional status"	
#16	#4 and #9 and #14 and #15	590
#17	"Animals" OR "Animalia" OR "Animal" OR "Metazoa"	383390
#18	#16 and #17	565

[5] Physiotherapy Evidence Database [PEDro]

1. For concept 'metastatic':
   TIAB: Exercise* and metasta* AND TI: cancer. Limited to clinical trials. 25 results
2. For concept 'advanced':
   TIAB: Exercise* and advanced and TI: cancer. Limited to clinical trials. 52 results.

[6] SPORTDiscus

Search	Query	Results
S1	TI (metastatic OR metastasis OR Metastases OR advanced OR advanced-stage OR palliative OR palliative care OR incurable OR End of life OR stage III OR stage IV OR inoperable) OR AB (metastatic OR metastasis OR Metastases OR advanced OR advanced-stage OR palliative OR palliative care OR incurable OR End of life OR stage III OR stage IV OR inoperable) OR KW (metastatic OR metastasis OR Metastases OR advanced OR advanced-stage OR palliative OR palliative care OR incurable OR End of life OR stage III OR stage IV OR inoperable)	13860
S2	TI (neoplasms OR neoplasm OR cancer OR carcinoma OR tumor OR tumour OR malignancy OR malignant OR solid tumor OR lymphoma OR leukaemia OR leukemia OR myeloma OR haematological malignancy OR hematological malignancy) OR AB (neoplasms OR neoplasm OR cancer OR carcinoma OR tumor OR tumour OR malignancy OR malignant OR solid tumor OR lymphoma OR leukaemia OR leukemia OR myeloma OR haematological malignancy OR hematological malignancy) OR KW (neoplasms OR neoplasm OR cancer OR carcinoma OR tumor OR tumour OR malignancy OR malignant OR solid tumor OR lymphoma OR leukaemia OR leukemia OR myeloma OR haematological malignancy OR hematological malignancy)	31257
S3	TI (Exercise OR exercise therapy OR exercises OR physical activity OR physical activities OR Endurance Training OR aerobic training OR Resistance Training OR resistance training OR strength training OR Weight Lifting OR weightlifting OR Physical Conditioning, Human OR physical conditioning OR Muscle Contraction OR Circuit-Based Exercise OR circuit training OR High-Intensity Interval Training OR interval training OR Running OR Jogging OR Bicycling OR Swimming OR Walking OR Sports OR sport OR Yoga OR Tai Ji OR tai ji OR tai chi) OR AB (Exercise OR exercise therapy OR exercises OR physical activity OR physical activities OR Endurance Training OR aerobic training OR Resistance Training OR resistance training OR strength training OR Weight Lifting OR weightlifting OR Physical Conditioning, Human OR physical conditioning OR Muscle Contraction OR Circuit-Based Exercise OR circuit training OR High-	593688

	Intensity Interval Training OR interval training OR Running OR Jogging OR Bicycling OR Swimming OR Walking OR Sports OR sport OR Yoga OR Tai Ji OR tai ji OR tai chi) OR KW (Exercise OR exercise therapy OR exercises OR physical activity OR physical activities OR Endurance Training OR aerobic training OR Resistance Training OR resistance training OR strength training OR Weight Lifting OR weightlifting OR Physical Conditioning, Human OR physical conditioning OR Muscle Contraction OR Circuit-Based Exercise OR circuit training OR High-Intensity Interval Training OR interval training OR Running OR Jogging OR Bicycling OR Swimming OR Walking OR Sports OR sport OR Yoga OR Tai Ji OR tai ji OR tai chi)	
S4	TX (body composition or muscle or fat or weight or BMI or sarcopenia or mass or lean or bone or dexa or DXA or Whole-body dual-energy x-ray absorptiometry or BIA or bioelectrical impedance assessment or cachexia or nutritional status or fat percentage or nutrition or Kg) OR KW (body composition or muscle or fat or weight or BMI or sarcopenia or mass or lean or bone or dexa or DXA or Whole-body dual-energy x-ray absorptiometry or BIA or bioelectrical impedance assessment or cachexia or nutritional status or fat percentage or nutrition or Kg)	387774
S5	S1 and S2 and S3 and S4	90

[7] National Rehabilitation Information Center Database [REHABDATA]

View Articles, including International Research, containing all of the words: cancer and exercise, containing at least one of the word(s): '"advanced" OR "metast*"', where Abstract contains: cancer, AND Abstract contains: exercise

Supplementary Table S2. Inclusion/exclusion criteria according to PICOS framework.

		**Examples for inclusion**	**Examples for exclusion**
**Population**	Patients with advanced or metastatic cancer (human subjects)	Metastatic OR advanced /'locally advanced'/stage III/IV<br><br>Mixed population but results of intervention in patients with advanced/metastatic cancer reported separately<br><br>If not reported separately, included if at least 75% of population has advanced/metastatic cancer	Animal models OR (non-metastatic AND non-advanced)<br><br>Only Stage I or II cancer<br><br>Fewer than 75% of patients advanced/metastatic cancer
**Intervention**	Exercise intervention (any type)<br><br>Nutritional intervention (any type) combined with exercise intervention (any type)	Any period of exercise training i.e. beyond a single session<br><br>Including physical activity/exercise advice/recommendation if adherence reported<br><br>Any nutritional intervention that is combined with exercise training	Nutritional intervention alone<br><br>Single session of exercise<br><br>Physical activity/exercise recommendations

			that do not report adherence<br><br>Electrical muscle stimulation without exercise<br><br>Vibration without additional exercise<br><br>Swallowing exercises<br><br>Inspiratory muscle training
**Control**	Comparison group	Usual (standard) care<br>Other intervention, e.g. exercise or nutrition intervention	Healthy controls<br><br>Non-metastatic/advanced patients
**Outcomes**	Body composition	Any measurement of the relative constituents of body mass, e.g.<br>Lean mass<br>Skeletal muscle mass<br>Fat mass	Undifferentiated total weight<br>BMI<br>Bone mass<br>Waist/hip circumference
**Study Design**	Prospective controlled trials	Randomized controlled trials<br>Non-randomized controlled trials	Observational studies<br>Retrospective studies<br><br>Studies with no control, e.g.<br>Single-arm studies
